# Multinodal Acoustic Trapping Enables High Capacity and High Throughput Enrichment of Extracellular Vesicles and Microparticles in miRNA and MS Proteomics Studies

**DOI:** 10.1021/acs.analchem.0c04772

**Published:** 2021-02-16

**Authors:** Axel Broman, Andreas Lenshof, Mikael Evander, Lotta Happonen, Anson Ku, Johan Malmström, Thomas Laurell

**Affiliations:** † Department of Biomedical Engineering, Faculty of Engineering, Lund University, 221 84 Lund, Sweden; ‡ Department of Clinical Sciences, Infection Medicine, Faculty of Medicine, Lund University, 221 84 Lund, Sweden; § Department of Laboratory Medicine, Faculty of Medicine, Lund University, 222 42 Lund, Sweden

## Abstract

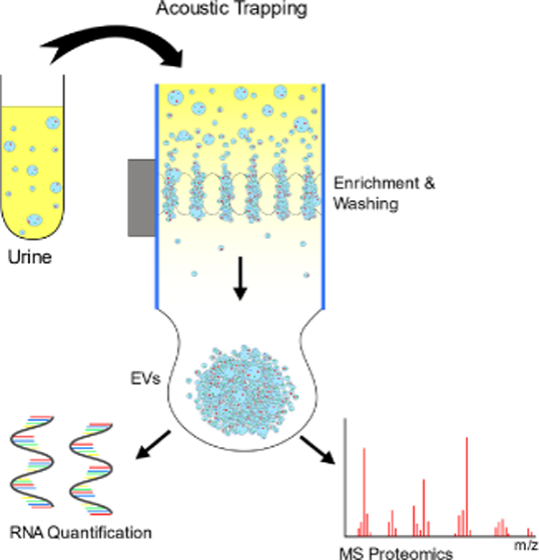

We report a new design of an acoustophoretic trapping device with significantly increased capacity and throughput, compared to current commercial acoustic trapping systems. Acoustic trapping enables nanoparticle and extracellular vesicle (EV) enrichment without ultracentrifugation. Current commercial acoustic trapping technology uses an acoustic single-node resonance and typically operates at flow rates <50 μL/min, which limits the processing of the larger samples. Here, we use a larger capillary that supports an acoustic multinode resonance, which increased the seed particle capacity 40 times and throughput 25–40 times compared to single-node systems. The resulting increase in capacity and throughput was demonstrated by isolation of nanogram amounts of microRNA from acoustically trapped urinary EVs within 10 min. Additionally, the improved trapping performance enabled isolation of extracellular vesicles for downstream mass spectrometry analysis. This was demonstrated by the differential protein abundance profiling of urine samples (1–3 mL), derived from the non-trapped versus trapped urine samples.

## Introduction

Extracellular vesicles (EVs) are small, membrane-enclosed particles that are released by cells and contain a wide range of bioactive molecules. These include proteins, lipids, and genetic information, often in the form of mRNA and non-coding RNA. EVs act as cell–cell messengers, shuttling around these bioactive molecules and help in regulating cellular function.^1^ Additionally, the content in EVs reflects the state of the parent cell and can therefore be used to assess the health and disease of the organism as a whole.^2,3^ As such, there is great interest in the study of EVs, which requires improved methods for isolating and enriching these vesicles.

The current gold standard for EV isolation is differential ultracentrifugation (UC), which is a laborious and time-consuming method that often requires large sample volumes. Additionally, ultracentrifugation frequently gives inconsistent results across studies due to the use of different rotors and variations in the protocols.^4−8^ Finally, the very large forces involved in UC may coalesce vesicles and form vesicle aggregates^9^ as well as coprecipitate larger protein complexes, e.g., Tamm–Horsfall proteins in urine.^10^

To overcome the problems with ultracentrifugation, many microfluidic devices for EV isolation and enrichment have been developed. These include nanoscale deterministic lateral displacement (nano-DLD),^11^ immunoaffinity-functionalized microstructures or beads,^12,13^ dielectrophoresis (DEP),^14^ viscoelastic separation,^15^ surface acoustic waves (SAW),^16^ and acoustic trapping.^17^ Microfluidic approaches rely on the deterministic laminar flow profile and have the benefit of working with much smaller sample volumes as compared to UC. Microfluidic devices have by nature a low throughput compared to conventional techniques. In the aforementioned methods for EV isolation, the flow rate is typically a few microliters per minute, without including any potential labeling or incubation time. This is inherently not a problem, as the volumes required for analysis can be quite small. It does however put an upper limit to the sample volume due to processing time. In the case of dilute samples like urine or cell culture medium, where larger volume needs to be processed, microfluidic approaches commonly fail.

Acoustic trapping is a promising technology for isolating and enriching EVs. It offers a gentle, label-free, non-contact way of capturing and retaining particles against a flow by generating a strong localized ultrasonic standing wave inside a microfluidic channel. The standing wave creates a stationary pressure node in the center of the channel, which can capture particles down to a few microns by the primary acoustic radiation force.^18,19^ The first demonstration to capture particles in the 100 nm range was reported by Hammarström et al.,^20^ utilizing scattered sound interaction from preloaded seed particles. Isolation of extracellular vesicles from blood plasma using acoustic seed particle trapping was first demonstrated by Evander et al.^17^ Numerous mass spectrometry-based proteomics studies have investigated EVs,^21^ and recently, Rezeli et al. were the first to demonstrate mass spectrometry-based proteomics data derived from microparticles isolated by acoustic trapping, although limited by the minute analyte amounts isolated by the trapping unit.^22^ Later, Ku et al. demonstrated the use of acoustic trapping to enrich EVs from biological fluids such as plasma, urine, and conditioned media for microRNA analysis. TEM images also verified an intact EV morphology after the acoustic processing.^23^ More recently, a SAW-based approach has also demonstrated the trapping of nanoparticles by means of scattered sound interaction with particles in a packed bed.^24^ Later, this system also reported EV isolation from cell culture supernatant at a throughput of 100 nL/min, indicating potential for significant scalability.^25^

In this paper, we present a novel, scaled-up acoustic trapping device with significantly increased throughput and capacity as compared to previously reported systems. The new device comprises a piezoelectric transducer and a glass capillary with a cross-sectional area 20 times larger than the capillary in the acoustic trapping system in the aforementioned studies. The capillary is actuated at a multinode resonance instead of the standard single-node resonance, generating nine trapping nodes instead of one. This enables more particles to be retained in the trapping zone, thus increasing the capacity of the device. Furthermore, since the ability to retain particles in the trap is dependent on the shear stress induced by the fluid flow, the larger capillary cross section allows for higher flow rates without the increased shear stress on the trapped particles. This now enables rapid, label-free processing of milliliter-sized samples, facilitating the acoustic isolation of EVs from dilute biological samples. In this paper, we therefore demonstrate that acoustic trapping using bulk actuation is scalable. Quantitative proteomics analysis revealed enrichment of distinct proteome patterns associated with the trapped urine samples compared to non-trapped urine, paving the way for future studies interfaced to MS-based proteomics or RNA sequencing analysis.

## Theory

### Radiation Forces

**Figure 1:**
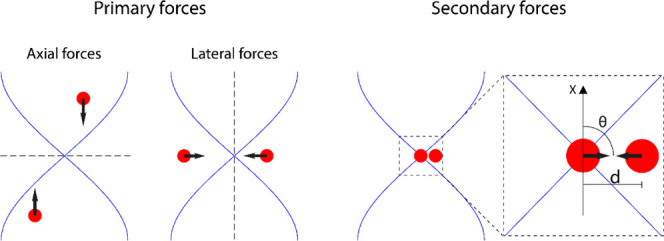
Summary of the acoustic radiation forces on particles with the positive acoustic contrast factor in an acoustic trap. The axial forces push particles into the nodal plane. Lateral forces, caused by the acoustic energy density gradient, push particles toward the center of the acoustic field and enable particle retention against the flow. Secondary forces become relevant when particle distances are small and cause particle aggregation. Illustration inspired by Hammarström et al.^20^

The theory behind acoustic radiation forces has been described by Gorkov,^26^ Whitworth et al.,^27^ Crum,^28^ Weiser et al.,^29^ and Groschl.^30^ Briefly, in acoustic trapping, a standing ultrasonic wave is generated inside a channel and will exert radiation forces on particles in proportion to the energy density in the resonator and the size of the particles as well as the particle density and compressibility relative to the surrounding medium. For a spherical particle in an ideal fluid, and if the wavelength is much larger than the particle, the magnitude of the force is equal to the average impulse flux through any closed surface of the sphere.^26^ The primary radiation force (PRF) of a plane standing wave on a particle with a radius much smaller than the wavelength is given by^31^
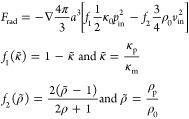
1where *a* is the radius of the particle, *f*_1_ and *f*_2_ are the monopole and dipole scattering coefficients, respectively, κ_p_ and κ_0_ are the compressibilities, ρ_p_ and ρ_0_ are the densities of the particle and the medium, respectively, and *p*_in_ and *v*_in_ are the pressure and velocity field time averages. For a one-dimensional planar wave, the expression is simplified to
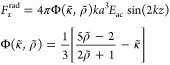
2where *E*_ac_ is the acoustic energy density, *k* is the wavenumber, and Φ is the acoustic contrast factor. The acoustic contrast factor indicates how a given particle behaves in a sound field. If the contrast factor has a positive value, the particle migrates toward the pressure node. If the factor has a negative value, the particle migrates toward the pressure antinode (Figure 1).

As part of the PRF, particles will also experience a lateral radiation force due to the fact that the sound wave is localized to the transducer region and the acoustic energy density diminishes rapidly outside the transducer area.^32^ The acoustic energy density gradient in the lateral direction (axial direction of the capillary) is perpendicular to the standing wave direction.^33^ The lateral radiation force is given by
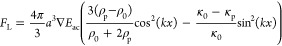
3Lastly, particles will also experience an interparticle radiation force that arises from sound scattering between particles in solution, commonly called secondary force.^30^ If the incident wave is a plane wave, the secondary radiation force on two identical particles at a distance *d* in a pressure field *p* is given as
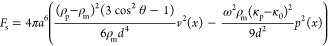
4

### Trapping Unit

**Figure 2:**
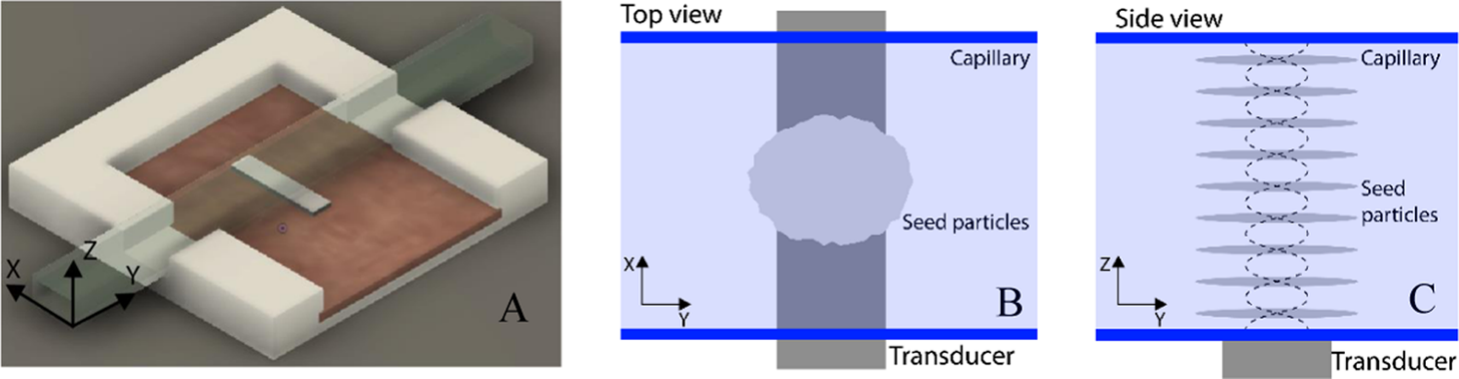
(A) Three-dimensional schematic of the acoustic trapping device. A glass capillary is attached to a piezoelectric transducer. The transducer is soldered to a circuit board for electronic interfacing and controlling the sound wave. (B) Model of the trapping device viewed from the top. Particles are trapped over the transducer. (C) Model of the standing acoustic wave inside the channel viewed from the side. Particle clusters are shown, collected in the nine nodes created by the standing wave.

The trapping unit consists of a borosilicate glass capillary (2 × 4 × 50 mm^3^) (Vitrocom) with connecting tubings, glued to an ultrasonic transducer (Pz26, Meggitt) and a temperature sensor (Pt100, Jumo) mounted on a printed circuit board (PCB), see Figure 2a. The sample is run through the capillary and a localized ultrasonic standing wave is generated inside the capillary in the region of the ultrasonic transducer.

The transducer is actuated at a channel resonance of 4.4 MHz. This results in a standing wave with nine trapping nodes along the height of the channel (Figure 2b,c). Particles are collected in these nodes and retained against the flow in the channel, enabling isolation, enrichment, buffer switching, and washing of the particles. Submicron particles can also be enriched in the trap by the secondary acoustic force by first loading the trap with large seed particles that interact with nanoparticles in close proximity. Using this method, particles of around 100 nm have been captured.^20^

The resonance frequency is dependent on the speed of sound in the medium and is therefore sensitive to temperature fluctuations and particles collecting in the trap. To compensate for small changes in the resonance frequency, a frequency tracking software developed by Hammarström et al.^34^ was used to continually update the signal generator frequency to match the resonance frequency of the trapping capillary. Briefly, the software periodically scans a frequency range around the resonance peak and measures the transducer impedance spectra. The software subsequently sets the output frequency to match the impedance minimum until a new scan is made.

## Experimental Section

### Measuring Trapping Capacity

The capacity of the trap was measured for five different actuating voltages (7, 9, 11, 13, and 15 V_pp_). A solution containing 12 μm polystyrene particles (Sigma-Aldrich) was run through the trap at 500 μL/min until the trap was saturated with particles. The capillary was then washed with 2 mL of Milli-Q water to remove any non-trapped particles. The retained particle cluster was then extracted by turning off the ultrasound and flushing the channel with 3.5 mL of Milli-Q at 5000 μL/min. Triton-X (0.1%) was added to the solution to mitigate particles sticking to the wall. Following vortexing and sonication, the solution was transferred to a BD Trucount tube and run through a cytometer (BD FACS Canto II) to count the number of particles.

### Measur**i**ng Throughput and Trapping Efficiency

The trapping efficiency of 500 nm polystyrene particles (PS) was measured at five different flow rates (100, 200, 500, 1000, and 2000 μL/min). The trap was first loaded with a seed particle cluster containing 12 μm polystyrene beads, and excess particles were washed away with 2 mL of Milli-Q at a flow rate matching the sample flow rate. Five hundred microliters of a solution containing fluorescent nanoparticles (Fluoro-Max 500 nm, polystyrene, Thermo Scientific) was then aspirated through the trap, followed by rinsing with 2 mL of Milli-Q to remove untrapped nanoparticles. The seed cluster, along with trapped fluorescent nanoparticles, was collected by turning off the ultrasound and flushing with 3.5 mL of Milli-Q at a flow rate of 5000 μL/min. The fluorescence intensity of the collected sample was then measured as the average reading from the trapped sample, aliquoted in four wells, using a 96-well plate reader (FLUOstar Omega, BMG Labtech, Germany) and compared to the input sample to calculate the trapping efficiency.

### Capturing Extracellular Vesicles from Urine Samples

We investigated the device’s capability of capturing EVs from urine samples. Urine from a healthy donor was centrifuged at 2000*g* for 10 min (Eppendorf Centrifuge 5702) to remove cellular debris, to prevent clogging the trap, and the supernatant was collected. The trap was mounted vertically, with the outlet pointing downward. The system was primed with Dulbecco’s phosphate-buffered saline (PBS), followed by loading the trap with seed particles (12 μm polystyrene). Different volumes of urine (1, 2, or 3 mL) were run through the trap at a flow rate of 500 μL/min to capture EVs. The trap was then rinsed with 1 mL of PBS to wash away the urine supernatant. Finally, the ultrasound was turned off and the cluster was allowed to sediment for 5 s to get closer to the exit before it was recovered in a volume of 250 μL of PBS at a flow rate of 5000 μL/min.

The samples were then analyzed either by nanoparticle tracking (NTA) (NanoSight LM14C, Malvern Panalytical, U.K.) or by chip-based capillary electrophoresis (Agilent 2100 Bioanalyzer System, Agilent). The NTA measurements assessed the size distribution and concentration of particles in the samples. The samples that were analyzed in the bioanalyzer were first treated with 0.5 μg/μL ribonuclease (RNase) to remove any free RNA, thus ensuring that any detected RNA originated from inside vesicles. Vesicle-borne RNA was then extracted using Norgen’s Single Cell RNA Purification Kit, following the protocol for “Total RNA purification from Plasma or Serum”. The RNA was eluted with 10 μL of elution buffer provided in the kit. One microliter of this solution was then loaded into an mRNA Pico Chip and analyzed in the bioanalyzer to give the length distribution and concentration of RNA in the sample.

### Capturing Extracellular Vesicles for Mass Spectrometry

To further assess the trap and to observe if there are clear differences in protein content between the trapped and non-trapped samples, the urine samples were analyzed using mass spectrometry. One, two, or three milliliters of urine was processed in the acoustic trapping unit following the protocol as above. The trapped and washed EVs from the acoustic trap, along with a triplicate of the non-trapped sample were lysed using a Bioruptor Plus (Diagenode) using 20 cycles (30 s on and 30 s off) using the low setting. The proteins in the samples were then prepared for quantitative data-independent acquisition mass spectrometry (DIA-MS) using trypsin double digestion. One hundred μL of each sample, along with 4.6 μL of a 10 M urea and 50 mM ammonium bicarbonate (ABC) solution and 2 μL of 0.5 μg/μL sequencing grade trypsin, (Promega) was mixed and incubated at 37 °C for 30 min. The urea-ABC solution (45.4 μL) was added and the samples were incubated at room temperature for 30 min. The cysteine bonds were reduced with 0.5 μL of 500 mM tris(2-carboxyethyl)phosphine (TCEP) (at 37 °C for 60 min) and then alkylated with 1 μL of 500 mM iodoacetamide (at room temperature for 30 min). The samples were diluted with 250 μL of 100 mM ABC to a urea concentration below 1.5 M, and 2 μL of trypsin was added for protein digestion (at 37 °C for 16 h). The samples were acidified to a pH of 2–3 using 10% formic acid and the peptides were purified using SOLAμ HRP reverse phase columns (Thermo Scientific). The peptides were dried in a SpeedVac (miVAC DUO) and reconstituted in 2% acetonitrile and 0.2% formic acid. The peptide content in each sample was measured using a spectrophotometer (DeNovix, DS-11 FX+) to ensure an equal amount of peptides from each sample (0.5 μg) was injected into the mass spectrometer.

### Liquid Chromatography Tandem Mass Spectrometry (LC-MS/MS)

The peptides were analyzed using data-dependent mass spectrometry analysis (DDA-MS) and data-independent mass spectrometry analysis (DIA-MS) on a Q Exactive HFX (Thermo Scientific) connected to an EASY-nLC 1200 (Thermo Scientific). The peptides were separated on a Thermo EASY-Spray column (Thermo Scientific 50 cm column, column temperature 45 °C) operated at a maximum pressure of 800 bar. A linear gradient of 4–45% acetonitrile in aqueous 0.1% formic acid was run for 50 min for both DDA and DIA. For DDA analysis, one full MS scan (resolution 60 000 for a mass range of 390–1210 *m*/*z*) was followed by MS/MS scans (resolution 15 000) of the 15 most abundant ion signals. The precursor ions with 2 *m*/*z* isolation width were isolated and fragmented using higher-energy collisional-induced dissociation at a normalized collision energy of 30. The automatic gain control was set as 3e6 for full MS scan and 1e5 for MS/MS. For DIA, a full MS scan (resolution 60 000 for a mass range of 390–1210 *m*/*z*) was followed by 32 MS/MS full fragmentation scans (resolution 30 000) using an isolation window of 26 *m*/*z* (including 0.5 *m*/*z* overlap between the previous and next window). The precursor ions within each isolation window were fragmented using higher-energy collisional-induced dissociation at a normalized collision energy of 30. The automatic gain control was set to 3e6 for MS and 1e6 for MS/MS.

### Mass Spectrometry Data Analysis

MS raw data were converted to gzipped and Numpressed mzML using^35^ the tool MSconvert from the ProteoWizard, v3.0.5930 suite.^36^ All data analyses were stored and managed using openBIS.^37^ DDA data acquired spectra were analyzed using the search engine X! Tandem (2013.06.15.1-LabKey, Insilicos, ISB),^38^ OMSSA (version 2.1.8),^39^ and COMET (version 2014.02 rev.2)^40^ against an in-house compiled database containing the *Homo sapiens* and *S. pyogenes* serotype M1 reference proteomes (UniProt proteome IDs UP000005640 and UP000000750, respectively), yielding a total of 22 155 protein entries and an equal amount of reverse decoy sequences. Fully tryptic digestion was used allowing two missed cleavages. Carbamidomethylation (C) was set to static and oxidation (M) to variable modifications, respectively. Mass tolerance for precursor ions was set to 0.2 Da, and for fragment ions to 0.02 Da. Identified peptides were processed and analyzed through the Trans-Proteomic Pipeline (TPP v4.7 POLAR VORTEX rev 0, Build 201403121010) using PeptideProphet.^41^ The false discovery rate (FDR) was estimated with Mayu (v1.7) and peptide spectrum matches (PSMs) were filtered with protein FDR set to 1% resulting in a peptide FDR > 1%.

The DIA data were processed using the OpenSWATH pipeline.^42^ For DIA data analysis, spectral libraries from the above DDA data set were created in openBIS using SpectraST (version 5.0, TPP v4.8.0 PHILAE, build 201506301157-exported (Ubuntu-x86_64)) in TPP.^43^ For DIA data analysis, raw data files were converted to mzXML using msconvert and analyzed using OpenSWATH (version 2.0.1revision: c23217e). The retention time (RT) extraction window was ±300 s, and *m*/*z* extraction was set at 0.05 Da tolerance. RT was calibrated using iRT peptides. Peptide precursors were identified by OpenSWATH (2.0.1) and PyProphet (2.0.1), using a false discovery rate of 1% at the peptide precursor level and 1% at the protein level, and TRIC^44^ for reducing the identification error. The resulting DIA data sets were analyzed using Jupyter Notebooks (version 3.1.1).

## Results and Discussion

### Multinode Acoustic Trapping

**Figure 3:**
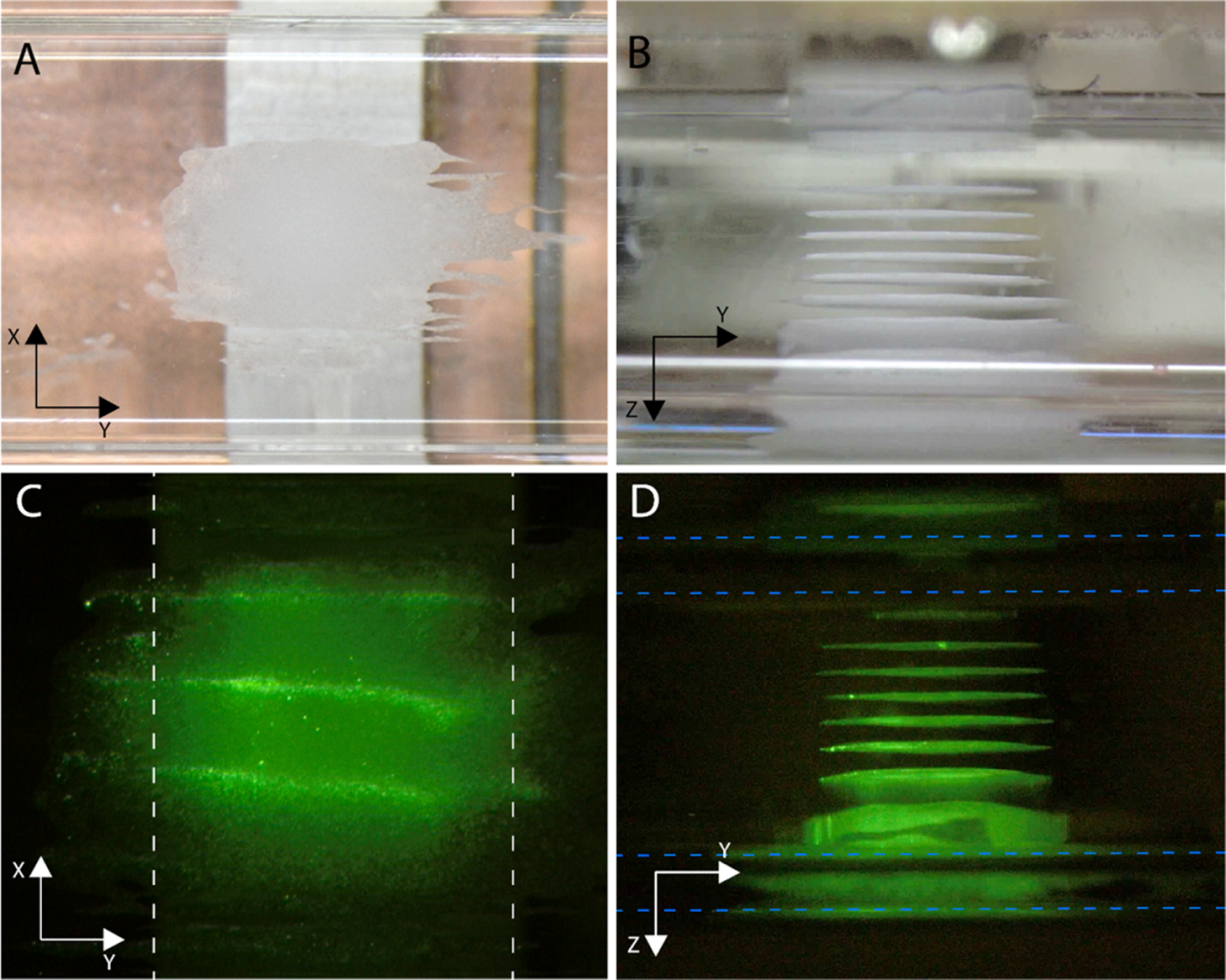
Pictures of particle clusters captured and retained in the multinodal acoustic trap. The flow rate in all pictures is 500 μL/min. (A) Bright-field image of the seed particle clusters viewed from the top. (B) Seed particle cluster viewed from the side. (C) Fluorescence image of a seed particle cluster enriched with 500 nm fluorescent polystyrene particles, viewed from the top. White dashed lines indicate the transducer. (D) Seed particle cluster enriched with 500 nm fluorescent polystyrene particles, viewed from the side. Blue dashed lines indicate the capillary wall. In both (B) and (D), nine clusters of particles can be seen stacked vertically above the transducer. The transducer is located at the top of the picture. The curved edges of the capillary cause optical distortions close to the wall, making the clusters appear more smeared.

The multinodal trapping unit can be seen in Figure 3. The trap showed nine distinct trapping nodes where seed particles (12 μm polystyrene) were enriched and retained against the flow (Figure 3a,b). Fluorescence imaging showed that it was also possible to capture and enrich smaller fluorescent particles (500 nm) (Figure 3c,d). It should be mentioned that pictures A, B, C, and D in Figure 3 were all taken of different clusters.

### Performanc**e** Testing with Polystyrene Beads

**Figure 4:**
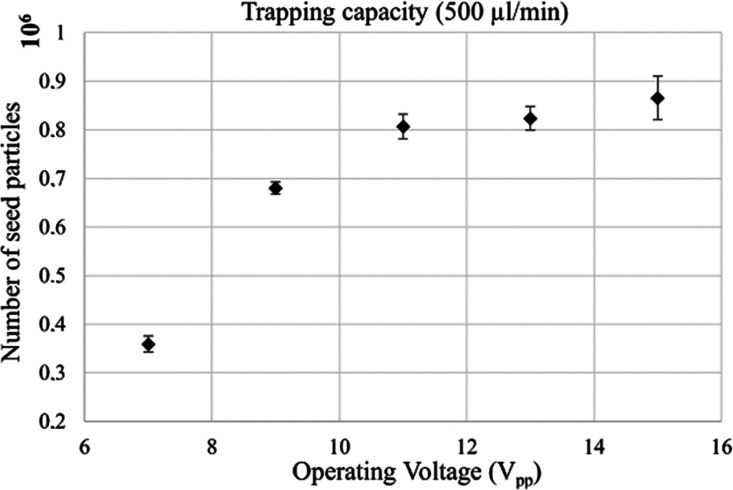
Capacity of the acoustic trap (actuated at voltages ranging from 7 to 15 V_pp_) as measured by the amount of 12 μm polystyrene seed particles that could be retained simultaneously at a flow rate of 500 μL/min. The standard deviation is displayed as error bars (*N* = 3).

To assess the trapping capacity of the device for different levels of input power, the ability of the multinode trap to retain seed particles at different actuation voltages was investigated. Increased power should increase the strength of the acoustic field but can also introduce problems with overheating. The system displayed increased trapping capacity with increasing voltage over the transducer (Figure 4), which is in agreement with the higher acoustic energy density in the trapping region at elevated voltage. However, the system saturated at around 860 000 seed particles (12 μm polystyrene) while operating at a flow rate of 500 μL/min (Figure 4). Compared to the commercial single-node AcouTrap system, which has a maximum capacity of 20 000 identical particles (data provided by AcouSort AB),^45^ this corresponded approximately to a 40-fold increase in the seed particle capacity of the system.

**Figure 5:**
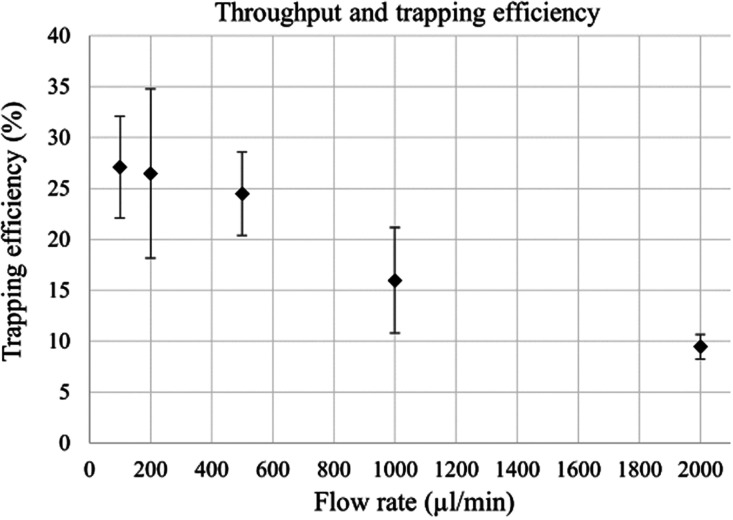
Trapping efficiency of the acoustic trap, as measured by the recovery of 500 nm fluorescent polystyrene particles, over a range of flow rates. The voltage was kept constant at 11 V_pp_ for all flow rates. The standard deviation is displayed as error bars (*N* = 3). The increasing flow rate decreases the trapping efficiency.

The throughput and trapping efficiency of the trap was evaluated by trapping fluorescent 500 nm particles at varying flow rates, with the trapping efficiency being defined as the percentage of input particles that are caught in the trap. The results from the throughput and trapping efficiency measurements can be seen in Figure 5. The large capillary allows for faster flow rates without increasing the drag force on the trapped particle clusters and without decreasing the time for the particle to migrate to the pressure nodes compared to a smaller capillary. The device was able to hold a stable seed particle cluster and trap submicron particles by the particle scattered sound interaction at flow rates of up to 2000 μL/min. The trapping efficiency decreased with the increase of the flow rate, with the highest average efficiency of 28% at 100 μL/min and the lowest of 9.5% at 2000 μL/min. The drop in trapping efficiency was expected as an increased flow rate increases the flow velocity of the particles and therefore decreases the time window for a given particle to be caught in the trap.

A high flow rate is a major advantage, as it allows for the rapid enrichment of particles and vesicles from larger sample volumes. It is clear that even though the multinode trap did not display a trapping efficiency higher than 28%, the high throughput still enables a rapid capture of particles/vesicles per unit time. This is highly useful for samples with a more dilute concentration of particles, for example, urine. An alternative to the multinode system presented herein could be multiple single-node trapping regions in series; however, the benefits of using a larger single trapping zone with multiple nodes become evident in the vastly increased flow rate offered. Furthermore, a system with multiple parallel trapping capillaries could possibly match the throughput of the multinode system, but the increased complexity in terms of driving electronics and associated costs of multiple trapping units as well as electronic circuitry makes it a less attractive alternative.

A flow rate of 500 μL/min was considered a good compromise between throughput and trapping efficiency and was chosen as the operating flow rate for further experiments with biological fluids.

### Extracellular Vesicles from Urine Samples

**Figure 6:**
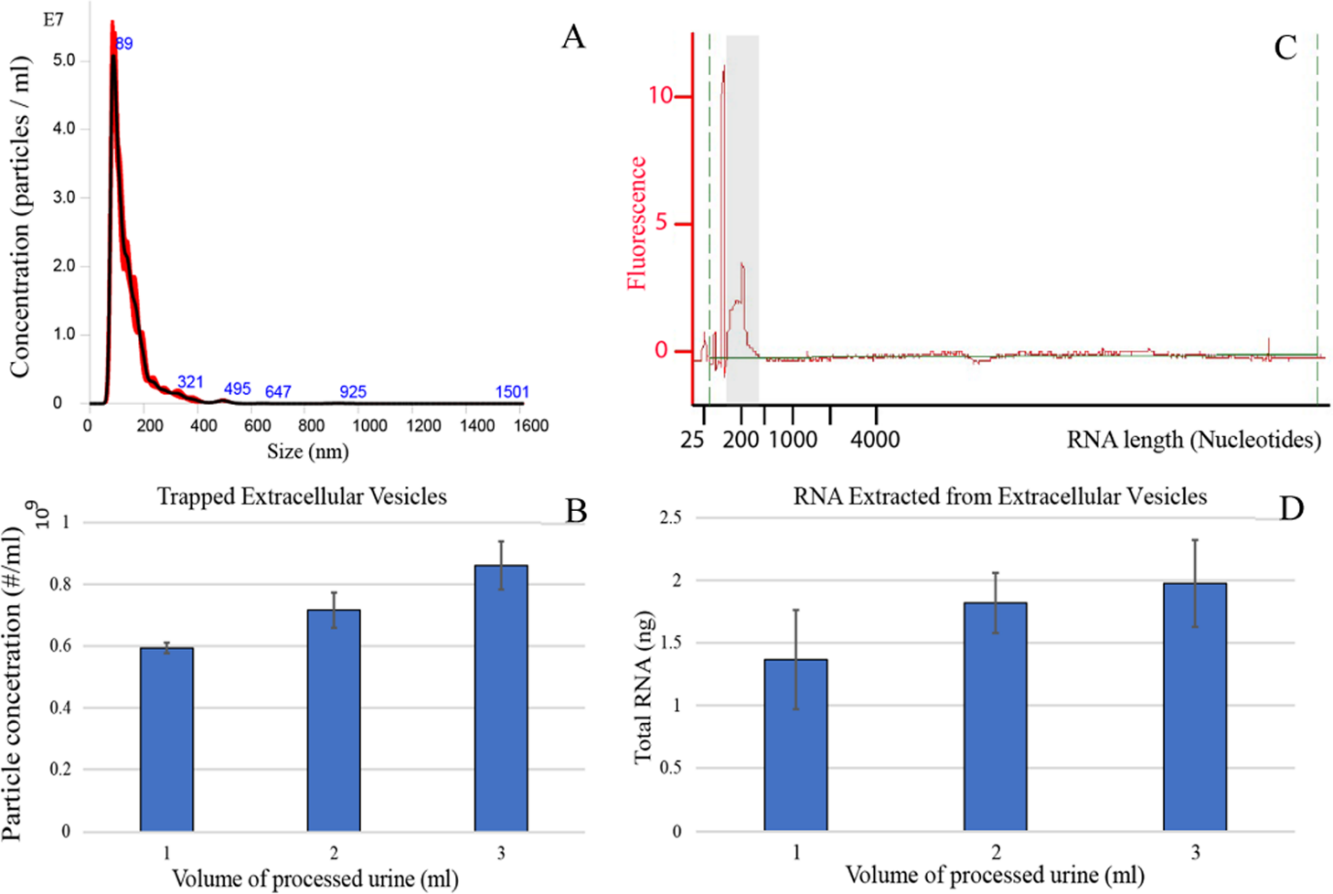
Extracellular vesicle trapping from 1, 2, and 3 mL of the urine samples. The flow rate was fixed at 500 μL/min for all samples. (A) The size distribution of particles from 3 mL of trapped urine with a vesicle peak at 89 nm. (B) Particle concentration in the eluted fraction of EVs from the NTA measurements for varying volumes of the input sample. Background from PBS has been subtracted. (C) The length distribution of intravesicle RNA from trapped vesicles expressed in the number of nucleotides. The first and the highest peaks represent the nucleotide reference ladder that was added to calibrate the system and should be ignored. The vesicle-derived RNA is seen in the peak around 200 nucleotides in length, highlighted in the gray zone. (D) The average total amount of RNA extracted for each input volume of urine.

After the trapping performance had been evaluated with 500 nm PS beads, we investigated the potential for trapping extracellular vesicles from the urine samples, using the optimized settings from above. Urine from a healthy donor (1, 2, and 3 mL) was processed in the trap and the particle content was evaluated using nanoparticle tracking analysis (NTA; Figure 6a,b), and the vesicle RNA content was measured using an Agilent mRNA Pico Chip in a bioanalyzer (Agilent 2100 Bioanalyzer System) (Figure 6c,d).

The NTA measurements showed a clear peak of EVs at 89 nm (Figure 6a), and the amount of trapped EVs increased with increasing input sample volume (Figure 6b). On average, 0.86 × 10^9^ EVs were trapped from 3 mL of urine. It can be observed that the sizes of the captured EVs were within the exosomal size range. The increase in trapped EVs was however not proportional to the input volume. Increasing the input sample from 1 to 3 mL only yielded a 45% increase in trapped EVs. An explanation for this could be that the trapping efficiency of particles might not be constant over time and we hypothesize that the system displays a higher trapping efficiency for the first fraction of a sample passing through the trap and as the seed trapping cluster fills with particles, the trapping efficiency drops correspondingly.

Further, Figure 6c illustrates the results of the bioanalyzer, with a peak in EV-derived RNA sequences at about 200 nucleotides in length, and Figure 6d gives the total amount of extracted RNA. As expected, processing a larger sample yielded an increased amount of intravesicular RNA. It should be noted that the samples were treated with RNase before the vesicles were lysed to eliminate any free-floating RNA from the samples. This ensured that all of the detected RNA originated from the internal vesicle cargo. On average, 3 mL of urine yielded 2 ng of purified intravesicular RNA. Similar to Figure 6b, the amount of recovered RNA did not increase proportionally with the increased input volume. Here, it is also seen that the increase in the input sample volume from 1 to 3 mL increased the RNA yield by 45%.

Our multinodal device trapped extracellular vesicles from urine at a flow rate of 500 μL/min. The total processing time for 3 mL of urine, including loading of seed particles, washing, and eluting, was 12 min and yielded 2 ng of intravesicular RNA. This can be compared with results from Ku et al.,^10^ who in a similar study, using a single-node system, isolated extracellular vesicles from the urine samples. They managed to isolate 0.79 ng of RNA from 9.75 mL of urine in approximately 20 h, operating at a flow rate of 15 μL/min and pooling EVs from 11 trapping rounds.

### Capturing Extracellular Vesicles for Mass Spectrometry

**Figure 7:**
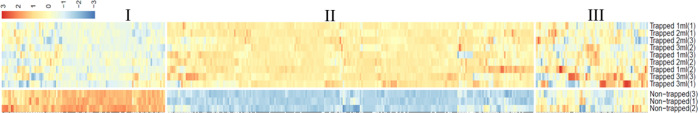
Heatmap of proteins found in trapped urine samples versus non-trapped urine samples. Keratin proteins have been removed. The heatmap is column-normalized and the legend gives the *z*-score of each sample. Cluster I shows weaker signals in the trapped fraction, suggesting that these are solute proteins that have been washed away from the trap during the washing step. Cluster II shows stronger signals in the trapped fraction, suggesting that these are proteins originating from vesicles that have been enriched during the trapping step. Cluster III shows highly fluctuating signals, indicating that these are proteins that are close to the limit of detection.

To evaluate how the enriched population of EVs differed in the protein content compared to non-trapped urine, we subjected the trapped and non-trapped samples to quantitative mass spectrometry analysis (Figure 7).

The subsequent MS analysis revealed substantial differences between the trapped samples and the non-trapped samples (Figure 7). In contrast, there were only minor differences in the protein content between the varying sample volumes. Visualizing the relative protein quantities in a column-normalized heatmap reveals three distinct protein clusters. Protein cluster I contains proteins found in higher abundance in the non-trapped samples, suggesting that these proteins are not associated with EVs and are washed away during the washing step of the trapping sequence. Proteins with stronger intensities in the trapped samples are found in cluster II and represent proteins that are associated with the EVs that have been enriched during the trapping step. Cluster III contains proteins with a higher degree of variability, due to partial or weak association with the EVs. The limited differences in the protein content for the different volumes of trapped urine are expected since the same amount of the starting material (0.5 μg) was injected for mass spectrometry analysis. Additionally, the data for each injection has been TIC-normalized (total ion current) to account for any differences in the amount of the peptide injected. Trapping more of the same urine sample should not change the type or ratio of the protein being captured; it should only change the amount of the protein captured. For a detailed heatmap where individual proteins can be seen, please see Supporting Information.

**Figure 8:**
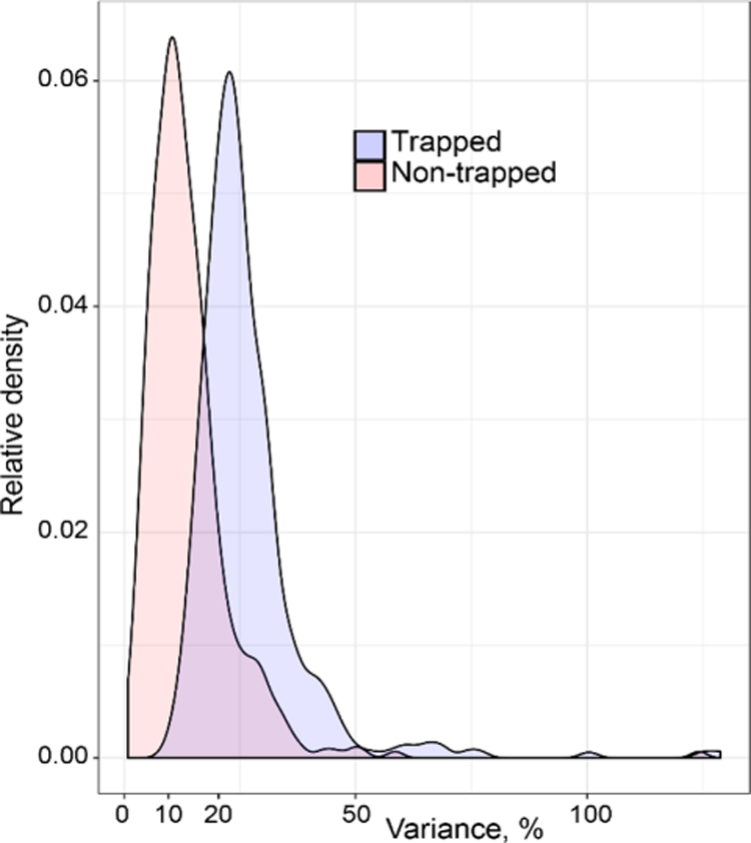
Variance of the content of individual proteins for the trapped samples and the non-trapped samples.

An important parameter for quantitative proteomics analysis is reproducible sample preparation. To investigate the degree of reproducibility, we plotted the relative standard deviation (RSD) for all proteins (Figure 8). The trapping resulted in a slight increase of the mean RSD from 10 and 20%. Importantly, the vast majority of the proteins have an RSD below 50%, which shows that the trapping only has a minor impact on reproducibility. We conclude that the sample preparation does not introduce a large increase in RSD, which is an important aspect for future quantitative proteomics comparisons for trapped EVs.

## Conclusions

In this study, we have presented a novel acoustic trapping device that supports a multinode resonance mode. Our results showed that the multinodal acoustic trap had significantly increased trapping capacity and throughput as compared to existing single-node systems. The multinode trap was able to capture extracellular vesicles in the exosome size range and could process 1–3 mL samples in the order of 8–12 min. The amount of isolated intravesicular RNA was in the low ng range. The MS proteomic analysis of proteins derived from the acoustically trapped samples displayed a significantly different protein expression profile as compared to the corresponding protein profile derived from the non-trapped urine samples. In comparison to other reports on microfluidic EV isolation, the throughput reported using multinodal acoustic trapping stands out. When comparing to more conventional EV isolation techniques, such as density gradient centrifugation, ultracentrifugation, ultrafiltration, immunoaffinity isolation, precipitation, and field flow fractionation, these fall short in the many hour-long processing times. An effort to present the performance of different EV isolation techniques was given by Wu et al.^46^ The new multinodal trapping system opens up for rapid, non-contact, and label-free EV isolation from biofluids that may pave the way for automated biomarker profiling in clinical samples.

## Supporting Information Available

Detailed heatmap comparing protein profiles in urine samples versus urinary EVs isolated by the new trapping platform (PDF)
Original MS data and a list of all identified proteins (XLSX)
