# Supplementary material for: Multinodal Acoustic Trapping Enables High Capacity and High Throughput Enrichment of Extracellular Vesicles and Microparticles in miRNA and MS Proteomics Studies
Source: Anal Chem. 2021 Feb 16;93(8):3929–37. doi: 10.1021/acs.analchem.0c04772 (PMC8023533; doi:10.1021/acs.analchem.0c04772)
Supplement: Supplementary file 1 — ac0c04772_si_001.pdf [file ac0c04772_si_001.pdf]

Supporting Information

Multinodal acoustic trapping enables high capacity and high throughput enrichment of extracellular vesicles and microparticles in miRNA and MS proteomics studies

Axel Broman[1*], Andreas Lenshof[1], Mikael Evander[1], Lotta Happonen[2], Anson Ku[3], Johan Malmström[2] and Thomas Laurell[1]

[1] *Lund University, Faculty of Engineering, Department of Biomedical Engineering, Sweden*

[2] *Lund University, Faculty of Medicine, Department of Clinical Sciences, Infection Medicine, Sweden*

[3] *Lund University, Faculty of Medicine, Department Laboratory Medicine, Sweden*

**Corresponding author: axel.broman@bme.lth.se*

Table of Contents

Figure S1. Detailed protein heatmap of trapped vs non-trapped urine samples

Table S1. List of proteins detected in urine samples.

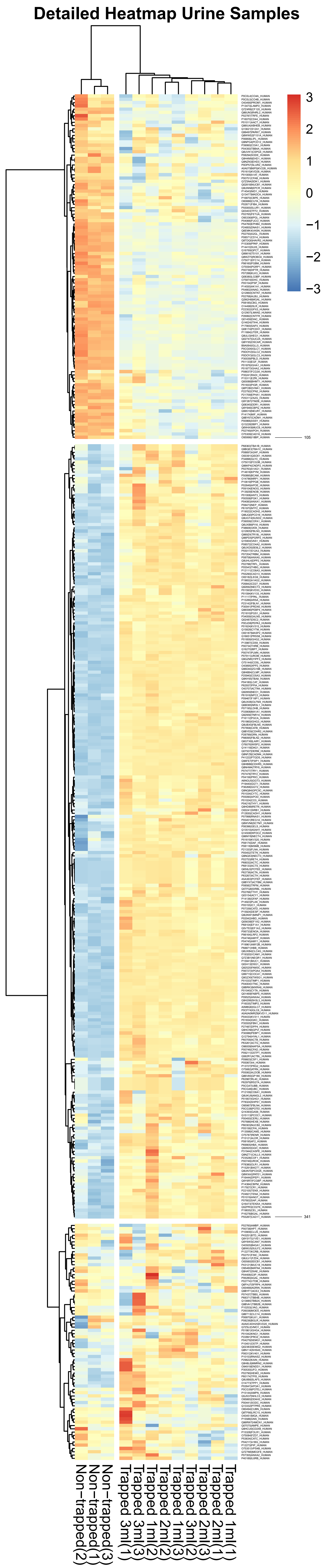

Figure S1: Detailed version of figure 7. Heatmap of proteins found in trapped urine samples versus non-trapped. Keratin proteins have been removed. The heatmap is row-normalized and the legend gives the z-score of each sample.

Table S1. List of proteins from heatmap in order from top to bottom.

Number	Uniprot ID	Uniprot Entry name
1	P0C0L4	CO4A_HUMAN
2	P0C0L5	CO4B_HUMAN
3	O43490	PROM1_HUMAN
4	P13473	LAMP2_HUMAN
5	Q7Z4R8	CF120_HUMAN
6	Q86UN3	R4RL2_HUMAN
7	P02787	TRFE_HUMAN
8	P16070	CD44_HUMAN
9	P01011	AACT_HUMAN
10	Q96IU4	ABHEB_HUMAN
11	Q13621	S12A1_HUMAN
12	Q99497	PARK7_HUMAN
13	Q8WW52	F151A_HUMAN
14	P06858	LIPL_HUMAN
15	Q9NPG4	PCD12_HUMAN
16	P39060	COIA1_HUMAN
17	P04350	TBB4A_HUMAN
18	Q6UVK1	CSPG4_HUMAN
19	P08294	SODE_HUMAN
20	Q9H4M9	EHD1_HUMAN
21	Q9NZN3	EHD3_HUMAN
22	P0DP57	SLUR2_HUMAN
23	A0A075B6P5	KV228_HUMAN
24	P01615	KVD28_HUMAN
25	P01009	A1AT_HUMAN
26	P00751	CFAB_HUMAN
27	Q7Z5N4	SDK1_HUMAN
28	Q02818	NUCB1_HUMAN
29	Q9UNN8	EPCR_HUMAN
30	P14543	NID1_HUMAN
31	Q13477	MADCA_HUMAN
32	P16870	CBPE_HUMAN
33	O60888	CUTA_HUMAN
34	P02671	FIBA_HUMAN
35	P55000	SLUR1_HUMAN
36	Q03403	TFF2_HUMAN
37	P02765	FETUA_HUMAN
38	O95336	6PGL_HUMAN
39	P04066	FUCO_HUMAN
40	P54760	EPHB4_HUMAN
41	P24855	DNAS1_HUMAN
42	Q6EMK4	VASN_HUMAN
43	P02750	A2GL_HUMAN
44	P08571	CD14_HUMAN
45	Q8TDQ0	HAVR2_HUMAN
46	P15309	PPAP_HUMAN
47	P14410	SUIS_HUMAN
48	Q16769	QPCT_HUMAN
49	Q99816	TS101_HUMAN
50	Q8WZ75	ROBO4_HUMAN
51	O75071	EFC14_HUMAN
52	P98160	PGBM_HUMAN
53	O75594	PGRP1_HUMAN
54	P00739	HPTR_HUMAN
55	P07288	KLK3_HUMAN
56	Q08380	LG3BP_HUMAN
57	O75874	IDHC_HUMAN
58	P05154	IPSP_HUMAN
59	P14550	AK1A1_HUMAN
60	P54802	ANAG_HUMAN
61	Q12860	CNTN1_HUMAN
62	P02768	ALBU_HUMAN
63	Q2M2H8	MGAL_HUMAN
64	P08185	CBG_HUMAN
65	O14498	ISLR_HUMAN
66	P22352	GPX3_HUMAN
67	Q12907	LMAN2_HUMAN

68	P26992	CNTFR_HUMAN
69	Q01459	DIAC_HUMAN
70	Q14624	ITIH4_HUMAN
71	P17900	SAP3_HUMAN
72	Q08174	PCDH1_HUMAN
73	P11684	UTER_HUMAN
74	Q9ULI3	HEG1_HUMAN
75	Q02747	GUC2A_HUMAN
76	Q8IYS5	OSCAR_HUMAN
77	B9A064	IGLL5_HUMAN
78	P0CG04	IGLC1_HUMAN
79	P0DOY2	IGLC2_HUMAN
80	P0DOY3	IGLC3_HUMAN
81	P30039	PBLD_HUMAN
82	P01133	EGF_HUMAN
83	P01876	IGHA1_HUMAN
84	P01877	IGHA2_HUMAN
85	P08637	FCG3A_HUMAN
86	P35241	RADI_HUMAN
87	P15311	EZRI_HUMAN
88	Q93088	BHMT1_HUMAN
89	P01833	PIGR_HUMAN
90	Q6PCB0	VWA1_HUMAN
91	P22792	CPN2_HUMAN
92	P21709	EPHA1_HUMAN
93	P25311	ZA2G_HUMAN
94	Q9Y287	ITM2B_HUMAN
95	Q08345	DDR1_HUMAN
96	Q9Y646	CBPQ_HUMAN
97	Q99519	NEUR1_HUMAN
98	P14174	MIF_HUMAN
99	Q9BY67	CADM1_HUMAN
100	P00966	ASSY_HUMAN
101	Q13228	SBP1_HUMAN
102	Q6W4X9	MUC6_HUMAN
103	P02749	APOH_HUMAN

104	O75309	CAD16_HUMAN
105	O95998	I18BP_HUMAN
106	P68363	TBA1B_HUMAN
107	Q9BQE3	TBA1C_HUMAN
108	P08697	A2AP_HUMAN
109	O00391	QSOX1_HUMAN
110	P34896	GLYC_HUMAN
111	O75015	FCG3B_HUMAN
112	Q96KP4	CNDP2_HUMAN
113	P02763	A1AG1_HUMAN
114	P14618	KPYM_HUMAN
115	P50895	BCAM_HUMAN
116	O14786	NRP1_HUMAN
117	P10619	PPGB_HUMAN
118	P02649	APOE_HUMAN
119	P09104	ENOG_HUMAN
120	P13929	ENOB_HUMAN
121	P01008	ANT3_HUMAN
122	P00558	PGK1_HUMAN
123	P04083	ANXA1_HUMAN
124	P08473	NEP_HUMAN
125	P61970	NTF2_HUMAN
126	P19022	CADH2_HUMAN
127	Q96JQ0	PCD16_HUMAN
128	Q9UGT4	SUSD2_HUMAN
129	P39059	COFA1_HUMAN
130	Q6UXB8	PI16_HUMAN
131	P38606	VATA_HUMAN
132	Q12805	FBLN3_HUMAN
133	Q969Z4	TR19L_HUMAN
134	Q96PD5	PGRP2_HUMAN
135	Q15904	VAS1_HUMAN
136	P08572	CO4A2_HUMAN
137	Q6UXD5	SE6L2_HUMAN
138	P55017	S12A3_HUMAN
139	P07204	TRBM_HUMAN

140	P08758	ANXA5_HUMAN
141	Q9UHL4	DPP2_HUMAN
142	P02788	TRFL_HUMAN
143	P05543	THBG_HUMAN
144	P12111	CO6A3_HUMAN
145	P55290	CAD13_HUMAN
146	O00182	LEG9_HUMAN
147	P19652	A1AG2_HUMAN
148	P26842	CD27_HUMAN
149	Q92692	NECT2_HUMAN
150	P01593	KVD33_HUMAN
151	P01594	KV133_HUMAN
152	P11117	PPAL_HUMAN
153	P15289	ARSA_HUMAN
154	P23142	FBLN1_HUMAN
155	P30041	PRDX6_HUMAN
156	Q96S96	PEBP4_HUMAN
157	P21810	PGS1_HUMAN
158	P34059	GALNS_HUMAN
159	Q02487	DSC2_HUMAN
160	P0DJD8	PEPA3_HUMAN
161	P01624	KV315_HUMAN
162	Q15828	CYTM_HUMAN
163	O00187	MASP2_HUMAN
164	Q16651	PRSS8_HUMAN
165	P01859	IGHG2_HUMAN
166	P13987	CD59_HUMAN
167	P00734	THRB_HUMAN
168	Q16270	IBP7_HUMAN
169	P00747	PLMN_HUMAN
170	P07911	UROM_HUMAN
171	Q9GZM5	YIPF3_HUMAN
172	O75144	ICOSL_HUMAN
173	O43895	XPP2_HUMAN
174	Q96DA0	ZG16B_HUMAN
175	Q9H6B4	CLMP_HUMAN

176	P25940	CO5A3_HUMAN
177	Q9NY65	TBA8_HUMAN
178	P04180	LCAT_HUMAN
179	P62937	PPIA_HUMAN
180	O43707	ACTN4_HUMAN
181	Q92859	NEO1_HUMAN
182	P61916	NPC2_HUMAN
183	P09467	F16P1_HUMAN
184	Q6UX06	OLFM4_HUMAN
185	Q68D85	NR3L1_HUMAN
186	P07195	LDHB_HUMAN
187	P33908	MA1A1_HUMAN
188	Q92956	TNR14_HUMAN
189	P16112	PGCA_HUMAN
190	P01860	IGHG3_HUMAN
191	Q9UBX5	FBLN5_HUMAN
192	P07858	CATB_HUMAN
193	Q9BYE9	CDHR2_HUMAN
194	P28799	GRN_HUMAN
195	P98095	FBLN2_HUMAN
196	Q6GTX8	LAIR1_HUMAN
197	O76076	WISP2_HUMAN
198	Q14118	DAG1_HUMAN
199	Q07507	DERM_HUMAN
200	Q8NFZ8	CADM4_HUMAN
201	P41222	PTGDS_HUMAN
202	Q96FE7	P3IP1_HUMAN
203	Q9HBB8	CDHR5_HUMAN
204	Q8NHM4	TRY6_HUMAN
205	P07477	TRY1_HUMAN
206	P07478	TRY2_HUMAN
207	P04156	PRIO_HUMAN
208	A6NGU5	GGT3_HUMAN
209	P19440	GGT1_HUMAN
210	P36268	GGT2_HUMAN
211	Q9NQ84	GPC5C_HUMAN

212	P01034	CYTC_HUMAN
213	P05090	APOD_HUMAN
214	P01024	CO3_HUMAN
215	P04216	THY1_HUMAN
216	Q9HD89	RETN_HUMAN
217	O00241	SIRB1_HUMAN
218	P12830	CADH1_HUMAN
219	P07998	RNAS1_HUMAN
220	P05451	REG1A_HUMAN
221	Q8WVN6	SCTM1_HUMAN
222	P06396	GELS_HUMAN
223	Q13510	ASAH1_HUMAN
224	Q14508	WFDC2_HUMAN
225	Q96NY8	NECT4_HUMAN
226	P01619	KV320_HUMAN
227	P08174	DAF_HUMAN
228	P08118	MSMB_HUMAN
229	P21333	FLNA_HUMAN
230	P05452	TETN_HUMAN
231	Q9NQS3	NECT3_HUMAN
232	P02753	RET4_HUMAN
233	P68032	ACTC_HUMAN
234	P68133	ACTS_HUMAN
235	Q6S8J3	POTEE_HUMAN
236	P62736	ACTA_HUMAN
237	P63267	ACTH_HUMAN
238	A5A3E0	POTEF_HUMAN
239	Q9BYX7	ACTBM_HUMAN
240	P08582	TRFM_HUMAN
241	Q5TFQ8	SIRBL_HUMAN
242	P02766	TTHY_HUMAN
243	Q03154	ACY1_HUMAN
244	P14136	GFAP_HUMAN
245	P14923	PLAK_HUMAN
246	P05155	IC1_HUMAN
247	P07339	CATD_HUMAN
248	P15924	DESP_HUMAN
249	Q9UNW1	MINP1_HUMAN
250	P02042	HBD_HUMAN
251	Q05639	EF1A2_HUMAN
252	P68104	EF1A1_HUMAN
253	Q5VTE0	EF1A3_HUMAN
254	P06733	ENOA_HUMAN
255	P98164	LRP2_HUMAN
256	P04746	AMYP_HUMAN
257	P04745	AMY1_HUMAN
258	P19961	AMY2B_HUMAN
259	P68871	HBB_HUMAN
260	Q6UXB4	CLC4G_HUMAN
261	P19320	VCAM1_HUMAN
262	Q7Z3B1	NEGR1_HUMAN
263	P15941	MUC1_HUMAN
264	Q02413	DSG1_HUMAN
265	Q92520	FAM3C_HUMAN
266	P06727	APOA4_HUMAN
267	Q99715	COCA1_HUMAN
268	Q9GZX9	TWSG1_HUMAN
269	P01033	TIMP1_HUMAN
270	P04004	VTNC_HUMAN
271	Q9BRK3	MXRA8_HUMAN
272	P01040	CYTA_HUMAN
273	Q01469	FABP5_HUMAN
274	P09525	ANXA4_HUMAN
275	Q9H299	SH3L3_HUMAN
276	P16035	TIMP2_HUMAN
277	A0M8Q6	IGLC7_HUMAN
278	P0CF74	IGLC6_HUMAN
279	A0A0A0MRZ8	KVD11_HUMAN
280	P04433	KV311_HUMAN
281	P01834	IGKC_HUMAN
282	P35555	FBN1_HUMAN
283	P27487	DPP4_HUMAN

284	Q9HCN6	GPVI_HUMAN
285	P30086	PEBP1_HUMAN
286	Q12794	HYAL1_HUMAN
287	P60709	ACTB_HUMAN
288	P63261	ACTG_HUMAN
289	O96009	NAPSA_HUMAN
290	P00746	CFAD_HUMAN
291	P09211	GSTP1_HUMAN
292	Q562R1	ACTBL_HUMAN
293	P09603	CSF1_HUMAN
294	P05067	A4_HUMAN
295	P13727	PRG2_HUMAN
296	O75882	ATRN_HUMAN
297	P05062	ALDOB_HUMAN
298	Q86V85	GP180_HUMAN
299	P62987	RL40_HUMAN
300	P62979	RS27A_HUMAN
301	P0CG47	UBB_HUMAN
302	P0CG48	UBC_HUMAN
303	P12109	CO6A1_HUMAN
304	Q9UKU9	ANGL2_HUMAN
305	P01857	IGHG1_HUMAN
306	P78324	SHPS1_HUMAN
307	O95967	FBLN4_HUMAN
308	P0CG38	POTEI_HUMAN
309	Q14393	GAS6_HUMAN
310	Q15113	PCOC1_HUMAN
311	P00450	CERU_HUMAN
312	P07686	HEXB_HUMAN
313	P80303	NUCB2_HUMAN
314	P05156	CFAI_HUMAN
315	P13598	ICAM2_HUMAN
316	O75787	RENR_HUMAN
317	P15121	ALDR_HUMAN
318	P08195	4F2_HUMAN
319	P69905	HBA_HUMAN

320	Q92820	GGH_HUMAN
321	P31944	CASPE_HUMAN
322	Q9NZT1	CALL5_HUMAN
323	P23528	COF1_HUMAN
324	P00749	UROK_HUMAN
325	P78380	OLR1_HUMAN
326	P15291	B4GT1_HUMAN
327	Q9UN70	PCDGK_HUMAN
328	Q8WXA2	PATE1_HUMAN
329	P16444	DPEP1_HUMAN
330	Q9Y6R7	FCGBP_HUMAN
331	P14384	CBPM_HUMAN
332	P17927	CR1_HUMAN
333	P22105	TENX_HUMAN
334	P24821	TENA_HUMAN
335	P01019	ANGT_HUMAN
336	P07602	SAP_HUMAN
337	Q16473	TENXA_HUMAN
338	Q5ZPR3	CD276_HUMAN
339	P19835	CEL_HUMAN
340	P16278	BGAL_HUMAN
341	P55287	CAD11_HUMAN
342	P02760	AMBP_HUMAN
343	P00738	HPT_HUMAN
344	P10909	CLUS_HUMAN
345	P43251	BTD_HUMAN
346	Q9Y5Y7	LYVE1_HUMAN
347	Q9Y6W3	CAN7_HUMAN
348	O43505	B4GA1_HUMAN
349	Q8IWU5	SULF2_HUMAN
350	P12277	KCRB_HUMAN
351	P02751	FINC_HUMAN
352	Q9ULV1	FZD4_HUMAN
353	O00560	SDCB1_HUMAN
354	P43121	MUC18_HUMAN
355	O95460	MATN4_HUMAN

356	Q9HAT2	SIAE_HUMAN
357	P04406	G3P_HUMAN
358	P06280	AGAL_HUMAN
359	P02774	VTDB_HUMAN
360	Q6FHJ7	SFRP4_HUMAN
361	O00468	AGRIN_HUMAN
362	Q9BYF1	ACE2_HUMAN
363	P07437	TBB5_HUMAN
364	P68371	TBB4B_HUMAN
365	Q13885	TBB2A_HUMAN
366	Q9BVA1	TBB2B_HUMAN
367	P10253	LYAG_HUMAN
368	P26038	MOES_HUMAN
369	Q86T13	CLC14_HUMAN
370	P06870	KLK1_HUMAN
371	P08236	BGLR_HUMAN
372	A0A0C4DH25	KVD20_HUMAN
373	Q7Z5L0	VMO1_HUMAN
374	P01861	IGHG4_HUMAN
375	P01042	KNG1_HUMAN
376	P22891	PROZ_HUMAN
377	P04279	SEMG1_HUMAN
378	P10451	OSTP_HUMAN
379	Q02383	SEMG2_HUMAN
380	Q8N114	SHSA5_HUMAN
381	P06312	KV401_HUMAN
382	P10153	RNAS2_HUMAN
383	P29622	KAIN_HUMAN
384	Q9H8L6	MMRN2_HUMAN
385	O94919	ENDD1_HUMAN
386	P30530	UFO_HUMAN
387	P02790	HEMO_HUMAN
388	P60174	TPIS_HUMAN
389	Q9UIB8	SLAF5_HUMAN
390	O14773	TPP1_HUMAN
391	P02647	APOA1_HUMAN
392	P0CG39	POTEJ_HUMAN
393	P15144	AMPN_HUMAN
394	Q5JS37	NHLC3_HUMAN
395	O95865	DDAH2_HUMAN
396	P00441	SODC_HUMAN
397	Q13332	PTPRS_HUMAN
398	O60494	CUBN_HUMAN
399	Q8TF66	LRC15_HUMAN
400	O43451	MGA_HUMAN
401	P15586	GNS_HUMAN
402	Q96RW7	HMCN1_HUMAN
403	Q07075	AMPE_HUMAN
404	Q9HCU0	CD248_HUMAN
405	P15328	FOLR1_HUMAN
406	O75084	FZD7_HUMAN
407	P53634	CATC_HUMAN
408	P04217	A1BG_HUMAN
409	P12273	PIP_HUMAN
410	O75351	VPS4B_HUMAN
411	Q7Z7M0	MEGF8_HUMAN
412	P07355	ANXA2_HUMAN
413	P40189	IL6RB_HUMAN
